# Survey of WBSNs for Pre-Hospital Assistance: Trends to Maximize the Network Lifetime and Video Transmission Techniques

**DOI:** 10.3390/s150511993

**Published:** 2015-05-22

**Authors:** Enrique Gonzalez, Raul Peña, Cesar Vargas-Rosales, Alfonso Avila, David Perez-Diaz de Cerio

**Affiliations:** 1 Tecnologico de Monterrey, Av. Eugenio Garza Sada 2501 Sur Col. Tecnológico, Monterrey, NL 64849, Mexico; E-Mails: raul.p.ortega@gmail.com (R.P.); cvargas@itesm.mx (C.V.-R.); aavila@itesm.mx (A.A.); 2 Technical University of Catalonia, C./Esteve Terradas 7, Castelldefels, Barcelona 08860, Spain; E-Mail: dperez@tsc.upc.edu

**Keywords:** wireless body sensor network, emergency mobile healthcare, optimization system for adaptive model, energy consumption

## Abstract

This survey aims to encourage the multidisciplinary communities to join forces for innovation in the mobile health monitoring area. Specifically, multidisciplinary innovations in medical emergency scenarios can have a significant impact on the effectiveness and quality of the procedures and practices in the delivery of medical care. Wireless body sensor networks (WBSNs) are a promising technology capable of improving the existing practices in condition assessment and care delivery for a patient in a medical emergency. This technology can also facilitate the early interventions of a specialist physician during the pre-hospital period. WBSNs make possible these early interventions by establishing remote communication links with video/audio support and by providing medical information such as vital signs, electrocardiograms, *etc.* in real time. This survey focuses on relevant issues needed to understand how to setup a WBSN for medical emergencies. These issues are: monitoring vital signs and video transmission, energy efficient protocols, scheduling, optimization and energy consumption on a WBSN.

## 1. Introduction

Life expectancy around the world has increased in the last years [1], which is good news, but it also implies an increase in medical care costs. For example, in Mexico, in 1930, people lived on average 34 years; 40 years later, in 1970, this indicator stood at 61, and by 2013, it was 76 years, [2]. In the same way, life expectancy in the U.S. has increased significantly; the number of adults between 60 and 80 years of age is expected to double in 2050 compared to the number registered in 2000 [3]. 

Life expectancy is not the only important factor for better medical care, e.g., traffic accidents also call for better and opportunistic emergency health services. Adequate and immediate medical assistance after an accident can minimize the possibilities of permanent injury or even death. Traffic accidents in Mexico are one of the main causes of injuries according to INEGI 2011, and are the second leading cause of death [4]. Table 1 shows the main external injury causes of death in Mexico in 2011. 

**Table 1:** Main indicators of external injury causes. INEGI, 2011 mortality rate per 100,000 inhabitants.

Type of Injury	Rate of Men	Rate of Women	Total Rate
Homicides	41	4.8	23.3
Traffic accidents	22.2	6.1	14.4
Suicides	7.8	1.9	4.9
Falls	3.2	0.8	2
Drowning	3.2	0.6	1.9
Burns	0.8	0.3	0.5
Poisonings	1.8	0.5	1.2
Total injuries	80	15	48.3

**Figure 1:**
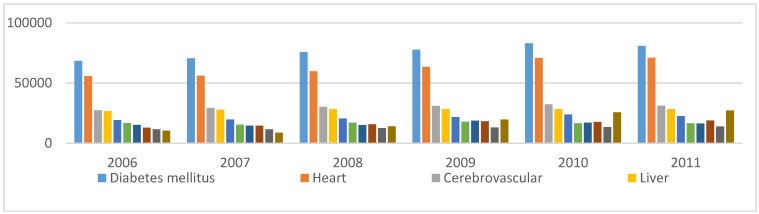
Main causes of death in Mexico, 2006–2011. Source: Death registry INEGI, 2011.

Figure 1 shows leading causes of death in Mexico from 2006 up to 2011. Traffic accidents and homicides are the leading causes of death that are unrelated to illnesses of internal organs. 

Therefore, availability of an efficient Wireless Body Sensor Network (WBSN)-based platform, capable of reporting data remotely to a medical center, is very important to increase the chances of survival. These networks are capable of transmitting data from images, vital signs and video. Information is acquired from sensors and cameras and upgraded through an electronic process for transmission and processing in a hospital using a friendly interface to the user. A platform example, is the *Emergency Remote Pre-Hospital Assistance* (ERPHA), a telemedicine system to deliver medical care to the patient at the location of an accident [5]. To successfully implement the ERPHA platform, the requirements for the provision of remote health services in real time are valuable information for the successful integration of mobile devices and wireless communication networks. 

### Emergency Scenario

Consider a WBSN with sensors reporting data remotely to a medical center through a mobile device such as a smartphone with video conference capabilities. Under a stable health condition, the Personal Health Information (PHI) of the patient should be reported to the medical center every 5 min. However, occurrence of an emergency event (e.g., traffic accident, natural disaster, heart attack, *etc.*) requires the reporting of PHI and video to be every 10 s, and the amount of data produced is incremented in a very short time. The scenario just introduced is an *emergency assistance scenario*, capable of saturating the WBSN, with different technological challenges including mobile computing, medical sensors, video technologies and communication for mobile healthcare (m-Health) systems [6].

**Figure 2:**
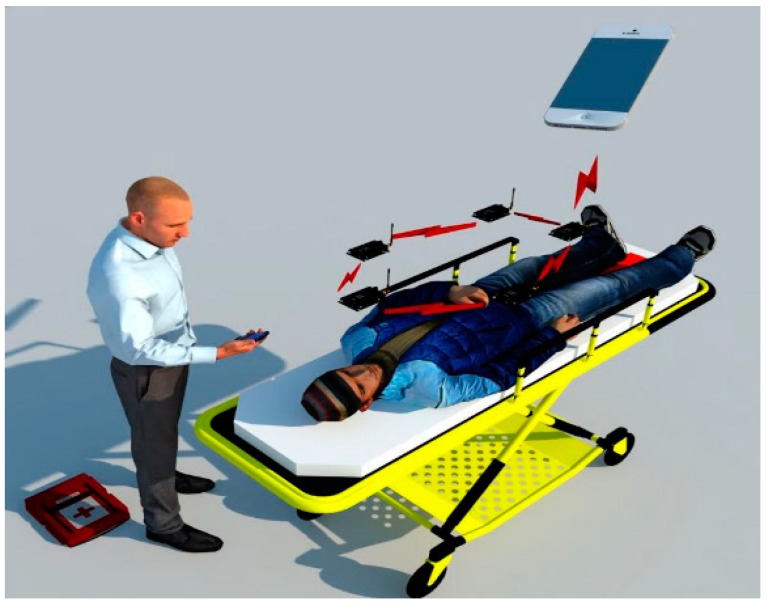
Wireless body sensor network communicating through a mobile phone.

In emergency situations, the integration of a WBSN and a smartphone can enhance the existing medical emergency procedures and minimize the possibilities of permanent injury or death. A basic WBSN-technology implemented in an emergency scenario is shown in Figure 2. It shows a body sensor network where a paramedic is connecting a patient. Sensors will communicate wirelessly to the mobile device and the mobile will capture parameters for subsequent delivery to the servers. Mobile networks help to reduce time and facilitate decision making in these circumstances.

Exploiting the potential benefits of these networks can result in additional challenging issues. One of these challenges is the interference which is generated by the coexistence of different technologies working in the same place. Interference may occur in crowded places such as airports, theaters, stadiums and hospitals. Other relevant issues to be considered are: scalability, sensor deployment, sensor density, energy efficiency, emergency detection and response [7].

Under an emergency scenario, the smartphone is going to experience both high energy consumption due to the execution of multiple tasks, and serious degradation in performance due to tasks such as a highly intensive monitoring and video streaming. Thus, the remaining energy in sensors of a WBSN becomes as critical as that on the smartphones. Although emergency scenarios are infrequent (*i.e.*, an average of 50 cases out of 10,000 [8]), these energy and performance issues represent an important technological challenge in terms of Quality of Service (QoS) assurance. In ZebranBAN [9], the authors addressed the efficient use of energy and performance improvement issues with the objective to support high-speed data transmission in future WBSN from the perspective of a heterogeneous network. 

This paper presents a survey of current trends in WBSN for emergency scenarios. Our paper also presents facts that play different roles for each medical scheme and have an impact on the network performance and the reduction of the WBSN lifespan. This paper also discusses issues about the medical emergency scenario such as WBSN architecture, recent studies in power efficient MAC protocols and the main communication protocols for m-Health. We also understand that efficient use of energy is the dominant factor in the design and implementation of protocols in wireless sensor networks for MAC/PHY and network layers in order to reduce power consumption and latency, and to increase throughput. An energy inefficient protocol may become the main source of energy waste [10]. Additionally, we introduce the fundamentals towards a definition of an optimization framework for the energy consumption on emergency scenarios. We also discuss communication and network protocols to improve performance of the WBSNs in terms of QoS. We also present relevant aspects such as: data compression and security of the medical information, and also the synchronization and joint transmission of this information with the video stream to enhance the network resources.

In Section 2, we present a review of previous work that addresses different components in a WBSN. Section 3 presents the vital sign evaluation, specific data features, signal compression, security on medical information, the WBSN architecture for emergency scenarios and relevant facts of Emergency E-Health practices. In Section 4, we analyze communication protocols, energy efficient MAC schemes and scheduling conflicts. In the fifth section, we discuss briefly the assessment of an energy optimization model for a WBSN. Section 6 presents the importance of video technology in m-Health systems. Finally, Section 7 and Section 8 present the future challenges and conclusions, respectively.

## 2. Literature Review

Previous surveys have concentrated on the analysis, discussion and global overview of WBSNs with the intention of orienting readers for future research work. It is important to say that there are many topics involved in the study of WBSN platforms, which makes difficult the standardization of ideas for the correct development of WBSNs. The authors in [10] presented a review of wireless sensor technology that divides sensor nodes into wearable sensors and implantable sensors, according to the type of signal acquired (glucose, blood pressure, oxygen saturation, temperature, *etc.*). The authors also indicated that power inefficient protocols are the main source of energy waste. Additionally, they presented an efficient routing protocol integrated with the MAC layer and indicated important implementation issues such as: security, authentication, data integrity, confidentiality, availability and privacy. 

Production of microelectronic chips with new sensing elements and systems for embedded data processing and energy harvesting are important for efficient implementation of WBSN applications [11]. Micek, *et al.*, in [11] are concerned with the study of energy efficient systems. The authors describe energy requirements, the subsystems of a WBSN node (sensing, data processing, control and communication subsystems), the strategies for data processing, a network topology through Cluster Heads (CH), routing methods, coding and modulation. The limited energy budget for WBSN applications makes necessary the visualization of an efficient use of energy. With respect to access methods, the authors mentioned the need to reduce collisions and transmit data with the minimum power and satisfying QoS parameters. Also, they discuss the challenges to minimize the transmission time, and the use of energy saving modes in network devices.

In [12], the authors discussed differences between Wireless Sensor Networks (WSNs) and WBSNs and presented an overview of physical layer properties, including common issues of communicating near or in-body. The paper also presented diverse types of devices with different data rates and energy consumption, as well as the need of quality of service, security and ease-of-use. With respect to energy consumption, it is suggested a combination of energy scavenging and lower energy consumption techniques as an optimal solution to have an autonomous system. The paper also discusses positioning of sensor nodes and its effect over radio wave propagation. Authors mentioned that radio signals may experience great losses due to absorptions of power in tissue, in the case of sensor nodes implanted inside the body. When nodes are located along the body, they divide the propagation of radio waves into: line of sight (LOS) and non-line of sight (NLOS) for testing. Contrary to the WBSN architecture discussed in [10], a two-tier architecture is introduced. The first tier is for the intra-body communications; the sensor nodes are attached to the body and a portable device collects data. The second tier is for communications between the portable device and the internet, and from the internet to a medical server. Furthermore, authors discussed the routing strategies for WBAN’s subdivided in two categories: routing based on the temperature of the body and cluster based protocols looking for a minimization of the energy consumption in the network. Finally, the authors pointed out the cross-layer protocols, as a way to improve efficiency.

In [13], the main body sensors are reviewed, with a discussion on selected physiological signals, typical network topology and data rates used for each type of sensor. It also introduces issues that affect physical layer protocols such as channel modeling, antenna design for in-body sensors and support for upper layer WBSN protocols for an appropriate balance between data rate, transmission distance and power consumption. Its authors propose a three tier architecture for health monitoring systems: (1) an intra-BSN tier, where body sensors collect physiological signals and then send data to a nearby personal server (PS) device; (2) an inter-BSN tier, which represents the communication between PS and one or more access points (APs); and (3) a tier for the use in metropolitan areas with a data base for medical record consulting and medical attention in real time through video conference, calls and text message by cell phones. Section 7 of [13] presents comparative information about some of the different BSN projects that have been conducted and a description of the diverse application areas (military, sports, healthcare) for WBSNs. Although [13] presents relevant information for BAN design, proposals to minimize energy consumption was not addressed.

A study of effective routing protocols in WBSNs is given in [14], where issues and challenges in terms of network topology, topological partitioning, body postural movements, short range transmissions, energy efficiency, and the overall network lifetime are addressed. The authors also present a discussion about limited resources, like Radio Frequency (RF) transmission range, poor communication capabilities, storage capacity and low bandwidth. 

In [15], the authors elaborate different application scenarios for healthcare and human-computer interaction, sensor/actuator devices, radio systems, and interconnection of WBSNs to provide a perspective on the trade-offs between data rate, power consumption, and network coverage. Also, authors presented a review of sensor devices, design characteristics and classifications (chemical, thermal, mechanical, and acoustic). The main contribution relies on an extensive review for the emerging and existing standard Radio Technologies for WBSNs and WPANs like Bluetooth Low Energy, UWB, Bluetooth 3.0, and Zigbee. This review also covers the open technologies like Insteon, Z-Wave, ANT, RuBee, and RFID. To apply these technologies, [15] points out that many issues remain to be addressed, one of those issues being the physical characteristics of sensor/actuator materials and electronic circuits. A second issue is the development and evaluation of improved propagation and channel models. A third issue is the management of resources and networking, and among other issues of importance are security, privacy, and power supply. A detailed investigation of the recent studies in WBSNs, challenges and results in the field from the perspective of the actual standard IEEE 802.15.6 is introduced in [16], with details on challenges and address allocation schemes for routing protocols; security requirements protocols and their energy consumption. A typical architecture of the system for a WBSN is divided into three tiers: Intra-WBSN, Inter-WBSN and Beyond-WBSN.

Finally, relevant studies have been conducted with the objective to optimize the BSN lifetime. In MEDISN [17], although some tests are done in hospitals, the coexistence between different technologies and noise signals that can be found inside a building is not evaluated. This evaluation is important to determine how much the network behavior will be affected using a deployment with multiple sensors operating at different transfer rates. With respect to the estimation of energy consumption in a sensor node, authors in [18] show a mathematical model that evaluates the power consumption of the network architecture based on the correct number of cluster heads participating in the network. 

Our objective is to give readers a relevant overview of important issues in WBSNs in order to set into motion the development of an optimization model. Literature reviewed in this section helps us to understand each part conforming a wireless system for medical care delivery. Unlike the articles reviewed in this section, in our consideration, the meaningful impact is first to recognize the relevance and the influence of the WBSNs application for medical care delivery, especially in emergency situations. The need for opportune and accurate parameters could very well depend on the optimization of the time parameters as in Section 5 is mentioned. With this model and after calculations, the system could be able to give an optimized solution allowing to adjust to the needs of real time monitoring together with the efficient use of energy in the WBSN. Additionally, in the reviewed articles, the utility of video technology for a WBSN platform is not particularly addressed. We consider that apart from physiological data, video technology is an important tool in emergency scenarios. Among that, the synchronization between physiological signals and video images of the patient could amplify the medical context from a specialist. In terms to efficient use of network resources, in Section 7, we open an idea to the joint transmission of physiological data and video.

## 3. Physiological Signal Monitoring and WBSNs Overall System

In this section, we present the elements and context of the m-Health emergency scenario. The transmission of vital signs from a remote place is a key feature to save lives in emergency events, also video streaming or videoconference support has become an important tool in emergency situations. In most cases, the paramedics who are the first to handle this events do not have the required expertise, with visual and audio support the specialist located at the hospital can give directions for proper patient care. Frequency, security and compression of the physiological signals are important for this purpose. We discuss briefly these issues. 

### 3.1. Vital Signs

Vital signs are those that make possible to evaluate the basic functions of the human being. The vital signs indicate the health condition of a person. The typical vital signs are:

- Electrocardiography (ECG): An electrical recording of the heart signal to detect anomalies.
- Electroencephalography (EEG): the electrical recording of the brain’s activity.
- Pulse: Measurement of the heart rate, that is, the number of times the heart beats during a determined period.
- Respiratory Rate: Inspiration and expiration activity within a specific time interval.
- Blood pressure: Pressure of the heart pushing blood, to distribute it against arteries resistance.
- Temperature: Measure of the body's ability to generate and get rid of heat.
- Oxygen saturation: Measure of the oxygen levels in the blood.

The testing frequency of the vital signs depends on the health condition of the patient. Inpatients should be checked for long periods of time, for example, a heart attack risk or post-surgery condition patient requires continuous 24-hour monitoring. For injured and stable patients, the test frequency is every hour. A table showing the different signal requirements of body temperature, pulse oxygen, blood glucose, blood pressure, ECG and EEG in a wireless body monitoring system, is presented in [19]. The sampling rates and data bits needed for each sample vary depending on signal characteristics and behavior. The sampling frequencies displayed in the table for body temperature, pulse-oximeters, and glucose are lower than ECG and EEG signals due to their slow change in time. The total transfer time per sample represents the amount of time required for each application to transfer information. Duty cycle comes from calculating the time ratio between active and inactive communication. Other values involved in the transfer of information within a wireless monitoring network as bit error rate (BER) and latency are shown in [20]. BER for vital signal wireless monitoring, is an important issue not at all defined by researchers. To set a BER limit desired for WBSN’s is not an easy task; there are important situations that could affect the reliability performance over the network as the distance between sensors and base station, number of sensors in the network, time intervals of each sensor, sampling rate, data packet sizes, body motion, environment surrounding the event, and if sensors are in line of sight (LOS) or not (NLOS). A work done in [21], mentions a BER of 0.002% as a reasonable rate for WBAN when the receiving device of a packet is in the line of sight of the sending node, while for non-line of sight (probably due to body motion) the value is set between 0.01% and 0.02%. The authors in [22] indicate that the BER increases significantly as the number on-body sensor nodes increases and an analysis performed shows that the number of sensor nodes in the WBAN must be limited to six in order to maintain an acceptable BER of 0.001. Tables shown in [19,20] specify information to choose relevant parameters involved in data monitoring, data collection and data transmission useful to provide feedback for system optimization.

### 3.2. Compression of Physiological Signals 

There are physiological signals with great demand in data collection and transmission rate, for example in [19], sample rates for ECG and EEG are set at 240 samples/s and 500 samples/s respectively. This amount of data is a major challenge in terms of battery life for wireless sensors. The power consumed by transmitting one bit wirelessly is at least 480 times the energy consumed by a simple 32-bit addition instruction; therefore, most of the battery energy is consumed in radio communications [23]. For m-Health services in an emergency scenario, major technological concerns are power battery, data transmission and limited network resources. Data compression is also a major concern and a growing trend under scenarios that collect, store, process and transmit huge amounts of data [24]. 

Compression has been used in WBSNs, with the most common method being wavelets. It is reported that this method can achieve nearly 80% compression in combination with neural networks [25]. For this study, an optimum threshold was used by considering an acceptable max absolute difference between the original signal and reconstructed signal less than 20. Another technique, based on predictive coding (a lossless compression technique), exploits redundancy between samples and has the advantage of reducing the total power consumption in the sensor nodes [26]. Although the actual compression techniques can achieve great reduction in data capacity and power consumption, there are still some challenges in compression technology techniques, e.g., to achieve a high level of data reduction without compromising the physiological signal integrity, and to develop methods that operate with a small processing power requirement. 

### 3.3. Security on Medical Information

Data security is a major problem for healthcare and medical emergency systems due to the handling of significantly delicate information. Sharing and transmitting private patient records via Internet can lead to interception of information by unauthorized parties. Security and integrity of the medical information is an important issue to be considered in the development of WBSNs. We briefly describe some important security considerations from the reviewed literature.

The Health Insurance Portability and Accountability Act (HIPAA) Security Rule was created for regulation of different kinds of medical services, and specifically focuses on the safeguarding of Electronic Protected Health Information (EPHI) [27]. The EPHI created, received, maintained, or transmitted by an entity must be protected against reasonably anticipated threats, hazards, and impermissible uses and/or disclosures. The security and privacy of patient-related data are two indispensable components for the system security of WBSN [28]. 

Information security can be seen from different perspectives such as privacy, integrity, and authentication. Privacy of patient’s data is an important issue of systems for healthcare. In terms of integrity, information needs to be intact and complete to ensure an accurate diagnosis. By authentication, the system must provide protocols where only authorized medical staff has access to information. In the emergency scenario, it is necessary to provide and define data security components due to the handling and transmission of patient information via public wireless networks, e.g., GPRS, 3G, 4G.

### 3.4. WBSN

Wireless personal area networks (WPANs) with intelligent physiological sensors allowing data acquisition and processing [29], and a wireless transmission system for disaster patient care have been proposed in [30]. Some of the existing challenges in a network with these features are: energy constraints, quality of service, sensor deployment, adaptability, real-time guarantee, network architecture, video transmission, among others [31]. A WBSN cannot have the same architecture as that of an environmental monitoring network, it should be different in order to maximize the life time of the network. Factors such as mobility of the patient, interference or noise, coexistence between communications protocols, features of vital signs, data rate, sampling, sensor duty cycle and security should be considered to develop an intelligent and adaptive WBSN for each scenario. There must be different strategies for sending data over a wireless network with reconfigurable topology. These strategies use parameters of sampling frequency, radio frequency, and data “priorities”, because some parameters are more critical than others. However, wireless communications within a WBSN have a major constraint: energy efficiency. Some factors that counteract the optimal use of energy are:

- Continuing and improper communication between devices.
- Housing resources and memory.
- Inefficient use of battery supply.
- Excessive traffic data and other types of information such as video and audio in real time.
- Interference and a large number of retransmissions.
- Inefficient network design that reduce power consumption of each node.
- Energy consumption of sensors according to the measured physiological parameter.

### 3.5. WBSN System Architecture

**Figure 3:**
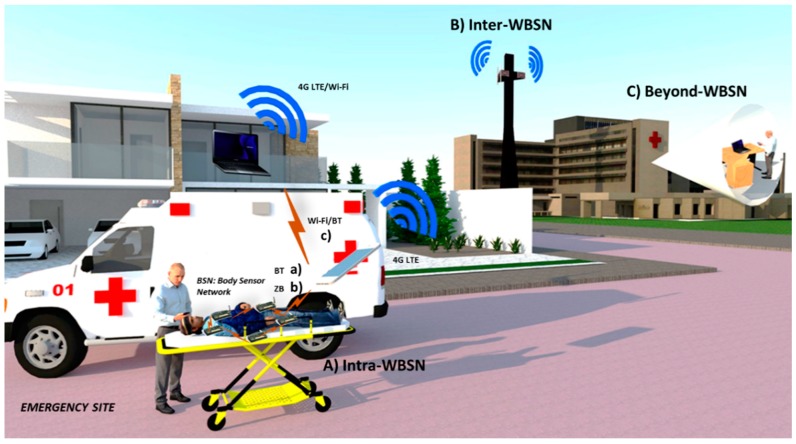
Wireless Monitoring Platform for Emergency Situations. (**A**) Intra-WBSN communication; (**B**) Inter-WBSN communication; (**C**) Beyond-WBSN communication.

Figure 3 illustrates a WBSN architecture for a health monitoring system, the emergency area can be divided into different disaster sites depending on the number of injured persons. Devices (e.g., PDAs, GPS, smartphones with video cameras, and vital sign sensors) could be managed by a local server. a) Sensors send data to smartphones by Bluetooth (BT), or b) ZigBee (ZB) technologies. Video and vital signs of patients are sent to a Relay Point (RP), which could be located in the emergency site or in the hospital. c) RP encodes and compresses the signals from each vital sign and sends them wirelessly (Wi-Fi or 4G) to a database server for record keeping and real time medical attention. Once data reaches the server, qualified staff will be able to manage the situation. For the emergency scenario, we integrate the WBSN communications architecture in three layers: Intra-WBSN communication, and Inter-WBSN communication, and Beyond WBSN communications [11].

- *Intra-WBSN Communication*: This layer considers communication between sensors and personal servers (usually less than 10 m away). Sensors have a direct wireless communication with PS through ZB or BT (star topology). The PS has capacity to take video of the patient’s condition, and collect and/or compress the physiological signals. The PS can directly transmit the video and physiological data, e.g., via 4G LTE to the second layer, but if the PS do not have this technology, the PS transmits the information to an ambulance via Wi-Fi or BT, and later the ambulance rebroadcasts the information via 4G LTE or Wi-Fi (modem enabled). According to [32], this tier should fulfill some hardware and software requirements. In terms of hardware, sensors must be lightweight, easy to use and comfortable for the patient, with high degree of accuracy. For the software, proper communication protocols, energy efficiency and handling of collected data are important considerations to make. Among these requirements, there are quality standards that must be implemented in the development of the system like: availability of data; usability (wearable or implantable); security/privacy, according to HIPPA; and QoS, for service guarantee.
- *Inter-WBSN Communication*: This tier corresponds to the communication between the PS to the internet or between PS and one or more RPs and then to the internet. If the signal drops, data could be sent to an RP/PC server (inside the ambulance) and afterwards, the information will be sent wirelessly (Wi-Fi) to the data base server. At this level, a gateway, PDA, PC, or 4G antennas are the link between de Intra-BAN and Beyond-WBSN layers. This tier needs some hardware and software requirements due to the specifications of the device, in terms of developing friendly user interfaces, and collecting and processing efficiently the sensors’ data in real-time [32].
- *Beyond-WBSN Communication*: This is the final layer which facilitates the real time medical attention. Medical staff could handle through internet video conference or mobile calls an emergency situation of an accident. With the help of a database server, all patient data collected will be stored to create the patient health profile, which could be consulted at any time needed. The inclusion of this tier depends on the application of the platform for a specific situation [32]. Some specifications are needed to implement the data server with respect to high capacity and processing, and should be located in the medical institution.

## 4. Energy Efficient Protocols, and Scheduling in WBSNs

Bluetooth and ZigBee can be used for data transmission between sensor nodes and PS, while Wi-Fi and 4G LTE technology are used for data transmission from PS to a medical center. In this section, we provide a brief descriptive comparison of existing communication protocols for WBSNs including Bluetooth Low Energy, ZigBee, Wi-Fi and 4G LTE, ANT+, NFC and Nike+, see Table 2. We also present medium access control or MAC which provides transmission channel access to multiple sensor nodes. The MAC layer is one of the most convenient options to achieve better energy efficiency in medical application networks. We start by discussing briefly the most common communication protocols in use:

- ANT+: It is an expanded version of the original ANT protocol to make the devices interoperable in a managed network. It was developed by the sensor company Dynastream in 2004 and it is considered a low power wireless technology. This protocol operates in the 2.4 GHz spectrum. ANT was created with the objective to communicate sports and fitness sensors with a display unit [33], which encourages its application in healthcare and telemedicine areas. Up to 64,000 members could participate in an ANT+ network; in ANT technology, the frequency channel is divided into many communication channels partitioned by time, allowing the sensors to remain in sleep mode for prolonged periods of time achieving important energy savings [34].
- NFC: NFC is for short-range wireless links operating at 13.56 MHz in the high-frequency (HF) band. His range of operation is approximately 4 cm and for this reason and its higher energy consumption compared with other low power wireless technologies (BLE or ANT+), it cannot be considered a direct competitor [34]. NFC involves two devices: an initiator (active) and a target (passive); the initiator actively generates an RF field that can power a passive target. Since passive targets do not require energy source, they can take different forms as tags, stickers, key fobs, or cards. In addition, peer to peer communication is possible, provided that both devices are powered (e.g., data exchange between two smartphones) [35].
- Nike+: It is a wireless technology created by Nike and Apple in 2006, (it will only work with devices of these brands) and operates in the 2.4 GHz spectrum supporting 250 Kbps or 1 Mbps transmission rates [36]. It was developed to monitor user’s physical exercise. Nike+ consists on a sensor attached to the body and a receiver, for example, a wrist band, watch or smartphone.
- ZigBee: It is a low power wireless protocol based on standard 802.15.4.2003, that was developed for applications that need network flexibility, security, high reliability, low cost, and simplicity. It is an *ad hoc* self-organizing network, and it was created for short range large-scale networks (over 65,000 for a ZigBee star network [37]) with low duty cycle and secure networking, but with smaller throughput needs and for low data rate applications. There are several covering areas for ZigBee such as: home automation, industrial, medicine, smart energy, telecom services, *etc.* For healthcare, ZigBee provides secure and reliable remote patient monitoring and management, with maintained freedom of mobility [33]. Some medical devices like glucometers, pulse oximeters, electrocardiographs and weight scales support ZigBee protocol.
- Wi-Fi: Wi-Fi is based on the IEEE 802.11 standards, letting users surf the Internet at broadband speeds when connected to an access point (AP) or in *ad hoc* mode. It is ideally suited for large data transfers, but unfortunately, it needs large batteries [33]. Wireless local area networks provide high-speed wireless connectivity and support information access anytime anywhere, allowing video-conferencing, voice calls, and video streaming. An important advantage is that all smartphones, tablets and laptops have Wi-Fi integrated, and the main disadvantage for Wi-Fi is high energy consumption even if its throughput is reduced.
- Long Term Evolution (4G LTE): 4G LTE is a technology that optimizes communications with transmission speeds of 100 Mbit/s for high mobility and 1 Gbit/s for low mobility. This technology uses transmission with Multiple Input-Multiple Output (MIMO) and Orthogonal Frequency Division Multiplexing (OFDM) techniques, transmitting more data in the same time period. On the negative side, 4G LTE is less power efficient than 3G and Wi-Fi for small data transfers, while for bulk data transfer is more power efficient than 3G [38].
- Bluetooth Low Energy (BLE): BLE was adopted and developed by the Bluetooth Special Interest Group aimed at novel applications in healthcare, fitness, security, and home entertainment industries [39]. The most important fact that guarantees Bluetooth presence in the market is its support by every major operating system. In the latest BLE improvement, a new stack protocol was proposed to offer energy efficiency, latency, piconet size, and throughput parameters. In the medical device area, BLE has the advantage that is implemented in majority of medical devices.

### 4.1. Energy Efficient Protocols in MAC Layer

Power efficiency is the dominant factor in the design and implementation of protocols in WBSNs, see Table 3. MAC protocols in WSNs aim to reduce power consumption and delay, as well as to increase throughput. Looking for energy saving medium access techniques, researchers have established two main approaches; Asynchronous: Low Power Listening (LPL); or Synchronous: Scheduled Contention (SC) for TDMA allocation. Nonetheless, TDMA schemes have limitations as overhead, lack of flexibility, adaptability and scalability. LPL and SC are scalable, flexible and adaptive but they are energy inefficient in transceiver mechanisms and overhead cost due to scheduling, respectively. Several mechanisms to optimize energy consumption in MAC protocols have been developed for WBSNs; an energy-saving Distributed Queuing (DQBAN) MAC superframe is introduced in [40]. The proposed DQBAN eliminates collisions and back-off periods in data packet transmission and behaves as a random access mechanism for low traffic load and switches smoothly and automatically to a reservation scheme when traffic load grows. DQBAN uses a superframe for communication between sensors to BAN coordinator with Contention Access Period (CAP) for body sensor access requests and an access Contention Free Period (CFP), exclusively for free collision transmissions.

**Table 2:** Main features of different communication protocols.

PROTOCOL	BLE	ZIGBEE	WIFI	4G LTE	ANT+	NFC	NIKE+
PROS	Continua Health Alliance technology (continuaalliance.org). Trade-off between energy consumption and bandwidth. In smartphones, sensors, tablets and laptops. Advanced encryption standard-128 algorithm.	Continua Health Alliance technology. Supports large-scale networks Transmits data over long distances. Low duty cycle.	Enabled in smartphones, tablets and laptops. The most efficient technology. Collision avoidance. High-speed wireless connectivity and allows communication between several devices.	High speed connectivity. Allows high definition video streaming. Mobility management system. Long range of connectivity.	Powered by a coin cell. Applications in healthcare and telemedicine. Sensors can remain in sleep mode for prolonged periods. Products from different manufacturers are interoperable.	The Passive component do not require energy source. Passive component can adopt simple forms [35]. Passive component can securely store personal data (e.g., credit or debit card information) [35].	Allow monitoring of physical activity. Powered by a coin cell.
CONS	Electromagnetic interference. Communication between only 2 devices at the same time. Cyclic sleep options not enabled. (but can be programmed)	Low Bandwidth (only 250 kbps). Existing smartphones. ZB doesn’t implement a coexistence scheme. Very few medical devices for sale	High energy consumption. Not suited for body sensors. Need to improve security. High cost technology.	High energy consumption, inefficiency of Periodic Transfers and overhead. Bad congestion Control and security. Large urban areas.	Compared with BLE, ANT+ is less adopted by devices. Low scalability due to random access transmissions [41]. High power consumption in master nodes compared with BLE [41].	Short range wireless link. Higher energy consumption compared with BLE and ANT+ Not enabled for healthcare Passive component contents data read-only.	Only works with Apple and Nike devices. Higher energy consumption compared with BLE and ANT+.
STANDARD RANGE [33,35,42]	1 to 100 m	10 to 100 m	~100 m	1–10 miles	~30 m	<0.2 m	~10 m
TOPOLOGY [33,34,42]	Star-bus	Mesh, Star, Point to Point	Star, Point to Point.	Ring, Star, chain, mixed.	Peer to Peer, Mesh	Point to Point	Point to Point
EFFICIENCY [33]	0.153 µW/bit	185.9 µW/bit	0.00525 µW/bit	0.011 µW/bit	0.71 µW/bit	Not specified	2.48 µW/bit
LATENCY [33,39,42]	<3 ms	<5 ms (beaconless mode, at 250 kbps)	1.5 ms	10 ms	Zero	Polled typically every second	~1 s
USES FOR WBSNs	Intra-BAN Communication: glucose, Blood pressure, SPO2, Temperature, Heart Rate, ECG, watches and smartphones	Intra-BAN Communication: glucose, Blood pressure, SPO2, Temperature, Heart Rate and ECG.	Inter-BAN and Beyond-BAN Communication: Smartphones, wireless modems, tablets, laptops.	Inter-BAN and Beyond BAN Communication: Smartphones, wireless usb modems, tablets.	Intra-BAN communication: Heart Rate, Temperature, Blood pressure, glucose and smartphones.	Not applications for BAN’s	Intra-BAN communication: Heart Rate, smartphones, wrist bands and watches.

When the communication is from BAN coordinator to body sensors, the BAN coordinator uses a feedback frame to synchronize the attached body sensors to the BAN coordinator. In addition enabled power management solutions and energy-aware radio activation policies among different time intervals is proposed with the intent to increase network lifetime. The protocol has been extended to have a fuzzy logic system in order to reduce scheduling collisions [20]. Each sensor should have a fuzzy logic system to deal with multiple cross-layer input variables of diverse nature in an independent manner. If the sensor node has the imperative need to send information since it has very low residual power or because the package to send has a significant delay, the sensor will be able to demand a “collision free” time slot. Also, the sensor can refrain from transmitting in the event that the sensor does not find the necessary conditions to do so (e.g., bad link channel). The implementation of a system like this offers the opportunity to optimize the MAC layer in terms of energy consumption and quality of service.

Another way to increase the lifetime of the network is through the use of the Lower Energy Adaptive Clustering Hierarchy (LEACH) protocol. It consists of introducing collection points in the network, *i.e.*, nodes receiving data from neighboring nodes, and nodes that perform data processing for subsequent transmission to the destination. In LEACH, the nodes organize themselves into local clusters, with one node as Cluster Head (CH), and each node determines its CH by choosing the CH that requires the minimum communication energy. Once all the nodes are organized into clusters, each CH creates a schedule for the nodes in its cluster. However, LEACH presents some problems at this collection points or CHs; a possible bottleneck is responsible for different tasks and data aggregation could be lost and never sent to the base station. A new improved LEACH protocol is developed to solve the potential problems in [43] which adds a Mediation Device (MD) node for all clusters responsible for synchronization between CH and its nodes. MD allows the CH to go to sleep mode periodically and to wake up only when it receives a wakeup signal from MD to receive data. Transmission of data during their time slots is based on a TDMA schedule. Once the CH has all the data from the nodes in its cluster, the CH node aggregates the data and then transmits the compressed data to the base station.

BODY MAC is proposed in [44], as an energy efficient MAC protocol with a frame structure, band allocation schemes and sleep mode as main tools. The MAC frame structure (TDMA-based) consists of three parts: Beacon, Downlink, and Uplink. Beacon is used for synchronization, and also contains network information that is broadcasted to nodes periodically. Downlink comprises the transmission (unicast or broadcast) from the gateway to the nodes. Uplink is divided in a Contention Access Part (CAP) and a Contention Free Part (CFP) for access to the channel. The duration of these parts is adjusted adaptively by the gateway according to the traffic characteristics. Band allocation schemes (Burst Bandwidth, Periodic Bandwidth and Adjust Bandwidth) are used by the gateway in order to decide how long the downlink and uplink periods should be and how to share the bandwidth resource. The use of a flexible band allocation improves energy efficiency by reducing the possibility of collisions and the overhead of radio transmission times, idle listening and control packets. The main energy is wasted when nodes have to stay awake to receive potential data. Better performance of BodyMAC protocol compared to that of IEEE 802.15.4 in energy consumption and end to end delay is obtained. 

**Table 3:** Comparison between different energy efficient MAC protocols.

PROTOCOLS	MAC OPERATION	ADVANTAGES	DISADVANTAGES	COMMENTS
BodyMAC [44]	TDMA	Flexible bandwidth allocation. Nodes and gateway synchronized allowing sleep mode. Good for periodic data sensing and event reporting.	Unsuitable scheme for collision avoidance.	Star topology and MICAz mote specification are used.
MEDMAC [45]	ADAPTIVE TDMA	Maximize energy efficiency through dynamical adjustments for QoS requirements. Good for low rate (Class 0) and medium data rate (Class 1) medical applications.	Low performance for high data rate applications.	Star topology. TDMA synchronization. Energy efficiency optimized by dynamically adjusting QoS requirements.
NEW IMPROVED LEACH [43]	TDMA/ Clustering	Distributed protocol, control information from the base station is not required. MD node is introduced to allow sleep mode periodically.	Extra overhead for dynamic clustering.	A clustering topology is used. Efficiency is increased 50% than LEACH protocol.
POWER EFFICIENT MAC [46]	TDMA/superframe enabled	Better performance in energy saving and delay compared to WiseMAC, ZigBee, B-MAC, and X-MAC protocols Wakeup mechanism enabled to reduce energy consumption with sleep mode.	No existing evaluation for security and QoS parameters. Nodes must wake up to receive the beacon.	Network lifetime is increased thanks to an overhead reduction. Two priorities for traffic: periodic or normal, and random or emergency.
HIGHLY RELIABLE ENERGY-SAVING MAC [20]	Distributed Queuing Body Area Network. Superframe is proposed	Qos parameters are considered. A Cross-layer fuzzy logic scheduler is used. By using Energy-aware radio-activation policies, sensors transmit at lowest possible level specified in 802.15.4.	Nodes must wake up to receive the beacon.	Star topology for BSN under two different realistic hospital scenarios. Matlab simulations are carried out using the CC2420 transmitter-receiver.
EQ-MAC [47]	Hybrid TDMA and CSMA schemes	Efficient node’s battery usage and support for QoS based on the service differentiation concept (data prioritization traffic levels).	Data redundancy in the sensor network. Low performance for low data rates. High latency without traffic prioritization.	Sensor Simulator is used for large-scale networks. EQ-MAC outperforms Q-MAC and S-MAC protocols.

The medical medium access control, MEDMAC in [45], is a TDMA based MAC protocol with some features such as: contention free channel access, and time slots dynamically adjusted. A Multi-Superframe is suited where a beacon period establishes the number of time slots from 2 to 256 including the contention free period and an optional contention access period. The synchronization mechanism introduces a Guard Band (GB) for each time slot to allow the sleeping mode of nodes between one or several beacon periods. The GB is calculated by an Adaptive Guard Band Algorithm (AGBA) so that each node has a dedicated time slot to avoid collisions. To compare MEDMAC with IEEE 802.15.4 MAC protocol, three types of data classes are mentioned:

- Class 0: Low grade data (e.g., respiratory, pulse and temperature sensor).
- Class 1: Medium grade data (e.g., ECG, EEG, blood pressure, Sp02).
- Class 2: High grade data (video, medical imaging, EMG, capsule endoscope).

Results indicate that MEDMAC can work more efficiently for low and medium data rate than IEEE 802.15.4 MAC. IEEE 802.15.4 has higher power consumption due to overhead; at medium grade data, MEDMAC performance is stable up to 24 nodes, while IEEE 802.15.4 is unstable for more than 13 nodes. Also, MEDMAC consumes less than 10% of the energy that IEEE 802.15.4 MAC protocol does.

An energy efficient MAC protocol using wake up radio is presented in [46]. Two basic priorities for traffic are defined: periodic or normal, and random traffic or emergency. WBSN devices are classified into full function device (FFD) and reduce function device (RFD). A Body Node (BN) can either be FFD or RFD and can respond accordingly to instructions received from the Body Network Controller (BNC). BNs are set in a sleep mode as a default state and the wakeup (managed by BNC) when they need to transmit or receive data (transition to idle state). A superframe with a beacon period and contention-free period (CFP) with 15 guarantee time slots (GTS) is advised. The communication process is divided in two phases: in the first phase BN receives the wake up radio signal from the BNC and verifies itself as the indicated receiver and sends an acknowledgement message; in the second phase, the main radio transceiver is triggered on and sends a beacon to guarantee the time slot for transmission. The behavior of BNC and BN are different depending on the priority of traffic. At normal traffic, BNC uses a table with wakeup schedule for every node where the wakeup interval is calculated from inter-arrival packets and the wakeup process is according to traffic intensity. When an emergency scenario (random traffic) occurs, BN wakes up and sends a wakeup signal to the BNC, then BNC acknowledges it and sends the beacon to the BN to allocate it into an available channel, finally BN sends its data to BNC. The evaluation of this MAC protocol demonstrates an increase in the network lifetime thanks to an overhead reduction. Better performance in energy saving and delay is found compared to WiseMAC, ZigBee, B-MAC, and X-MAC protocols. 

An energy efficient and QoS (EQ-MAC) protocol is presented in [47]. This protocol introduces a hybrid approach of scheduled TDMA and contention-based Carrier Sense Multiple Access (CSMA) schemes. It shows as advantage the use of service differentiation, handling highest priority packets to be processed immediately over those of the lowest priority. In terms of energy consumption and efficiency, EQ-MAC adapts better with high data rates, resulting in less energy compared with the standards S-MAC and Q-MAC protocols. Also, the energy consumption is not affected by the change in priorities. In terms of average packet delay, EQ-MAC achieves better performance under high priority data traffic, and in terms of delivery ratio, EQ-MAC adapts avoiding packet collisions by using scheduling of nodes. In conclusion, prioritizing traffic is a key aspect to improve performance with minimum delay and with energy efficiency.

### 4.2. Minimizing Scheduling Conflicts

The reduction of energy consumed for communications between sensors is treated in [48]. A scheduling technique for sensor nodes is introduced in order to allocate each node in a specific timeslot. Authors suggest spreading each round (period of time) on some slots. Knowing that a slot is the amount of time needed by a sensor to receive data from another sensor, they propose to wake up a sensor at specific times. Pairs of sensors will become active in order to perform at a specific time slot the communication of data. At the end of such time slot, a new pair of sensors will communicate while the previous one will go into sleeping mode until all the sensors have used a time slot which is called a round. At the end of each round, a relay sensor will be able to aggregate the data collected for its transmission to the base station. Since at every time slot only two sensors could communicate, collisions are avoided. The scheduling is adapted in order to cope with network changes due to sensors leaving or joining, so the authors concluded that the adaptive scheduling will allow decreasing energy consumption.

Inefficient use of battery because of retransmissions, data loss, high latency and excessive traffic data are some of the issues that we could face if scheduling conflicts for sensor transmissions are not resolved. An efficient scheduling design brings a very important reduction in power consumption of each node. An intelligent scheduling algorithm must be applied to allow the maximization of efficiency in performance, acceptable packet delay levels, and minimum power consumption. An example would be to form a characteristic vector for each sensor [49], as:

{I, RB(t), RE(t), RRi}
(1)

where I is the Indicator function (Abnormal Data); RB(t) is the Buffer space remaining; RE(t) represents the Energy remaining and RRi is the Importance of the physiological parameter. With the values of each of these variables, the vector is compared among sensors that are struggling to transmit at the same time. Comparison starts by the RRi variable, the second variable would be “I”, as it may indicate an abnormal vital sign measurement on patients and is considered of high importance, followed by RB(t) to avoid loss of information due to lack of space in the buffer, followed by RE(t). The above terms are compared until a sensor with higher priority is identified and its transmission is allowed over the next period. Another way to find a solution to the problem is to implement a scheduling algorithm, which integrates a fuzzy logic system in each sensor to deal with multiple input variables that allows each node to decide to transmit or not in the next time slot [20]. 

## 5. Energy Consumption and System Optimization in WBSNs

WBSNs need to carry out processes that are energy efficient, therefore, it is important to develop a model of energy consumption that is suitable to be optimized. The variables of the model can be compared according to the needs and behavior of the WBSN at any given moment (adaptive algorithm). We consider having a WBSN with *N* sensor nodes and a base station, which is able to compress, gather and process information received from each node in the network. The nodes are able to wirelessly communicate among themselves and also with the base station [18].

Nodes in the WBSN have mainly three different subsystems to operate, the first for vital parameter measurement, the second for sensor data recording, and the third for data sensor communications. Considering tradeoffs between latency and power consumption, we can formulate a model that can be used for performance optimization of the WBSN. Since energy efficiency is a main concern, the objective function represents the energy consumed in the WBSN. The decision variables of the optimization problem are the time intervals for each of the *N* sensors to carry out their sensing and communication tasks. For this, we consider that a sensor *I =* 1, 2, ..., *N*, consumes $E_{SS_{i}}\left(b\right)$ energy in the sensing activity during which a one packet of *b* bits of length is produced. It also consumes $E_{reg_{i}}\left(b\right)$ energy for reading and storing into memory those *b* bits of information, and it consumes $E_{tx_{i}}(b,d_{ij})$ energy for the transmission or reception of a packet of *b* bits of length in a link with sensor *j* with separation distance $d_{ij}$. $E_{sh_{i}}$ denotes the energy consumed by each sensor node's state transition (active mode or sleep mode). The function that quantifies the energy consumed in the entire *N* sensor network, *i.e.*, *E* [18,49], is given by:

(2) $$E=\overset{N}{\underset{i=1}{\sum }}\left[E_{ss_{i}}(b)+E_{reg_{i}}(b)+E_{tx_{i}}(b,d_{ij})+E_{sh_{i}}\right]$$

Table 4 provides an expression for each of the energy consumption terms in Equation (2). All the terms are based on the concept of energy as the product of power and time duration. We define $V_{inp}$ as the voltage supply, $T_{ss}$ as the time needed for sensing and producing one bit of information, $I_{ss}$ as the sensing current for one bit of duration. We also define $I_{wr}$ as the current used during the storing process of one bit of information, $T_{wr}$ as the time needed to store such bit of information, $I_{rd}$ as the current used during the reading of one bit of information produced and $T_{rd}$ as the time needed to read one bit of information. $bE_{circ}$ is the energy consumed by the transmission or reception of *b* bits of information, *n* is the path loss exponent of the environment, and $E_{pamp}$ is the energy consumed by the power amplifier. Finally, $T_{A}$ is the active time of the sensor; $C_{N}$ < 1 is referred to as Duty Cycle; *I_A_* represents the active mode current consumed; $I_{S}$ is the sleep mode current consumed.

**Table 4:** Energy consumption according to different subsystems of sensor node *i*.

Operation	Energy Consumption
Data sensing	$E_{ss_{i}}(b)$ = $bV_{inp}I_{ss}T_{ss}$
Data register	$E_{reg_{i}}(b)$ = $bV_{inp}\left(I_{wr}T_{wr}+I_{rd}T_{rd}\right)$
TX and RX	$E_{tx_{i}}(b,d_{ij})$ = $bE_{circ}+bd_{ij}^{n}E_{pamp}$
State shifting	$E_{sh_{i}}$ = $T_{A}V_{inp}[C_{N}I_{A}+\left(1-C_{N}\right)I_{S}]$

From the subsystems belonging to each node, the highest energy consumption is in the subsystem responsible for the transmission and reception of data. The term $E_{pamp}$ cannot be optimized since the energy expenditure in the power amplifier, is determined by the manufacturer and will only vary depending on the distance between the network components trying to communicate. As described previously, and based on a convex optimization model [49], only $bE_{circ}$ will be evaluated for the optimization as follows:

(3) $$bEcirc_{i}\;=\;\left(Ptx_{i}SF_{i}M_{i}\right)/DR\;+\;(Ptx_{i}\cdot O_{v})/\left(DR\cdot T_{i}\right)$$

where $Ptx_{i}$ is the transmission power; $SF_{i}$ represents the sampling frequency of sensor *i* (samples per second); $M_{i}$ is the length of measurement sample in bits per sample, data rate is defined by *DR*; $O_{v}$ and $T_{i}$ are used to describe Overhead and Data updating time interval of sensor *i*, respectively.

### 5.1. Constraints 

Based on a patient's condition and the level of monitoring required, the physician can specify a monitoring interval of $T_{idr}$ data for each sensor update, *i.e.*, the time interval for sensor *i,* will satisfy 0$\;\leq T_{i}\leq T_{idr}$ for i=1, 2…, N. There is a restriction regarding the total amount of available time partitions. Each partition or "slot" has a *T* size, which gives 1/*T* partitions in a time interval unit. Since each sensor is 1/*T* partitions in this interval, and each partition can be assigned at most to one sensor, we get the restriction $\overset{N}{\underset{i=1}{\sum }}\frac{1}{T_{i}}\leq \frac{1}{T}$ [49]. Sample size measurement between two updates will be constrained to only a fraction $X_{i}$ of the size of buffer $B_{i}$, without exceeding $X_{i}B_{i}/\left(SF_{i}M_{i}\right)$. Another constraint is referring to the total bits to transmit by each update, which is limited to the Time Slot length *DT*, Data Rate and the Overhead $\left(\frac{DT\cdot DR-Ov}{SFiMi}\right)$.

### 5.2. Cost Function

The cost function is composed of energy consumption and latency. The energy consumption of sensor *i* in a time interval is given in Equation (3), which can be simplified into [49]:

(4) $$bEcirc_{i}=\;E_{0}+bEcirc_{i}T_{i}$$

where the first term $E_{0}$ will remain constant at each time interval $T_{i}$, it will only vary from node to node depending on the vital sign that is being measured. The second term based on the overhead protocol, is the variable component. The other cost function component is the latency, *L,* which is the time it takes a sensor between collecting a sample and transmitting it to the base station and is given by:

(5) $$L=\overset{N}{\underset{i=1}{\sum }}\beta_{i}\frac{T_{i}}{2}$$

The final cost function is given by:

(6) $$C_{i}=\alpha_{i}bEcirc_{i}+\;\beta_{i}\lambda L_{i}$$

where $\alpha_{i}$ corresponds to the relative weight assigned to the power consumption in sensor *i*, $\beta_{i}$ is the coefficient indicating higher priority in order to minimize latency $L_{i}$ of sensor *i*, and λ is to give importance to latency relative to energy consumption term. The final optimization problem based on the cost function and the constraints is expressed as follows:

(7) $$\overset{N}{\underset{i=1}{\sum }}\left(\alpha_{i}bEcirc_{i}+\;\beta_{i}\lambda L_{i}\right)$$

Constrained to:

$$\text{0 }\leq T_{i}\leq \text{min}\left(T_{ir},\frac{X_{i}B_{i}}{SFi.Mi},\;\frac{DT\cdot DR-Ov}{SFiMi}\right),$$

$$\overset{N}{\underset{i=1}{\sum }}\frac{1}{T_{i}}\leq \frac{1}{T}$$

The optimization problem considering the research work done in [18,49], is the basis to develop an adaptive optimization system, capable to evaluate the circumstances of monitoring required due to network conditions, patient’s condition and the application scenario.

## 6. Video Technology in M-Health Systems 

Video technology continues to enhance safety and effectiveness in medical care. For emergency cases, the use of wireless video transmission or video conferencing is expected to have a significant impact on the procedures to be taken by the paramedics during the first hour (golden hour) after the accident. The specialist can provide instructions to paramedics based on video images of the victim and his physiological parameters, to sustain a better treatment that can have positive implications for the patient’s survival [50,51].

Furthermore, it can be of critical importance to the specialist to be aware of the situation of the victim prior to arrival at the hospital. Video transmission continues to be more efficient thanks to the advances in wireless networks and video compression technology. One of the emerging video coding standards is H.264/Advanced Video Coding (AVC) which has been developed and standardized collaboratively by both the International Telecommunication Union (ITU-T) and the International Organization for Standardization/ International Electrotechnical Commission (ISO/IEC) [52]. 

The H.264/AVC standard is built on the concepts of MPEG-2 and MPEG-4, and it offers the potential for better efficiency, *i.e.*, better quality compressed video, and greater flexibility in compressing, transmitting and storing video. The fact that the specialist from the hospital could have a video image of the situation of the victim from the accident, e.g., to see his face color, his reactions, his injuries, helps to a faster treatment when the patient arrives to the hospital, where the specialist is already prepared. Thus, the use of video is a significant step for the emergency medical care scenario. 

### 6.1. Video and Physiological Data Platforms

The literature includes a variety of platforms for m-Health support systems. In one, the standard H.264/AVC has been applied to telemedicine support situations of ambulance transferring emergency patients, where Ubiquitous Health (U-Health) is currently utilized without breaking medical law. Remote Medical Support System (RMSS) used in ambulances enables a doctor at a remote place to identify the patient status through video, and supply remote support to the emergency [53]. Another application is a mobile tele-care system installed in moving vehicles to support emergency cases, which provides videoconference and vital sign transmission services in real time [54]. The integration of the videoconference is an important support tool for a specialist located in the hospital to maintain visual inspection of the patient at a remote place.

The transmission of medical data traffic and video over the network, includes an adaptive rate scheme for multimedia medical data (video, ECG, medical scans) multiplexing in order to reduce the required resources from the telemedicine application [55]. The quality of service (QoS) through a step-by-step improvement of HSPA for m-Health services (video, ECG, vital signs, file transfers) to manage and meet the requirements for different scenarios is presented in [56]. It can be seen that the transmission of data and video is a common implementation in the architecture of a medical care system in an emergency scenario. Therefore, the inclusion of audio/video technology in a WBSN is needed to ensure the successful care of the patient with a mobile health system.

### 6.2. Video and Physiological Signal Synchronization

The combination of data and video plays an important support role. Improvements in the health delivery process in the context of tele-health requires the synchronization of video and physiological signals to make possible for the physicians located in a Hospital to simultaneously observe the face and the vital signs of the patient located at the place of the accident. Also, effective procedures for medical treatment, diagnosis, feedback and research require the synchronization of both video and biomedical signals. Performing feedback procedures to enhance patient safety in anesthesia operating rooms requires the creation of a permanent and accurate record of clinical events containing synchronized video, audio and vital signs for future offline analysis [57]. Procedures for diagnosing sleep breathing disorders need the synchronization between video recordings and polysomnographic readings to confirm breathing anomalies in pediatrics [58]. Identification, classification and quantification of silent neonatal seizures needs synchronized recordings of video and EEG signals to determine the correlation between the electrical and clinical artifacts in premature babies. Clinical manifestations recorded in video such as lip smacking, fixing of eyeballs or cyclic movement of legs are correlated with EEG recordings during diagnosis procedures [59]. 

The biomedical signals being synchronized with the video are usually more than one to provide sufficient medical and visual information to the physician. This information supports the physician in improving the accuracy and effectiveness of medical treatment, diagnosis and feedback. Observing one or more biomedical signals simultaneously with the video of the patient is necessary for assessment and correlation. Many signal- and image-processing challenges lie in the joint processing and transmission of biomedical signals and images. We see challenges in three areas: (i) multi-modality signal synchronization; (ii) joint signal, image and video compression; and (iii) interactive collaborative environments. There are significant synchronization issues for joint decoding. Clearly, one-dimensional biomedical signals should correspond to video images. As an example, we note the synchronization of ECG, respiratory, two-dimensional, and Doppler signals in ultrasound systems. In addition, we note that two-way voice communications must be synchronized to all clinical signals, as well as to real-time video images of the patient and the doctor. Resynchronization in the presence of wireless-communication errors will require innovative error-resilience methods. For well-synchronized signals and images, methods for joint-signal, image, and video compression can be developed [50].

## 7. Discussion and Open Research Issues

The specific technical challenge is to study, analyze and optimize the behavior of energy consumption in a wireless sensor network for medical monitoring. It is necessary to increase efficiency per node, in other words, a greater data transmission per node with respect to a quantity of energy available. It would be relevant to establish different strategies for data transmission over a wireless network with reconfigurable topology. These strategies will use the parameters of the sampling frequency, frequency for sending data, as well as the "priorities" of data to send. There are parameters that are more critical than others in a WBSN, therefore the priority in sending data from each sensor is different, e.g., in an emergency situation or critically ill patients. A model of analysis that carry out energy consumption estimations in sensors and personal servers must be considered to create a low energy consumption algorithm for transmission and reception of data with the aim to increase the battery lifetime in a WBSN. Some energy conservation challenges in WBSN must be attained to reach a reliable performance:

1. *Scheduling*: Design a transmission scheduling for the WBSN in relation to the transfer rate of each vital sign. Characteristics of a model in monitoring and data transmission must be provided. We know that sampling frequency of each sensor, required bandwidth and amount of data are very particular according to the vital sign measured.
2. *Adaptability*: A WBSN must be dynamic and adaptive. Each patient according to his/her condition, shall imply different priorities in terms of the energy consumption, the collection and transmission of data from each sensor.
3. *Communication*: wireless communication is an important factor to evaluate. Each sensor can communicate wirelessly with other sensors or with the base station, however, there are factors that can influence this communication to be successful to optimize energy consumption:
  a) Collisions if two sensors try to transmit at the same time (inefficient communication).
  b) Retransmissions caused by collisions or loss of information with direct impact on the energy consumption.
  c) Mobility of patient: when a patient is moving within different areas in a hospital (Emergency room, laboratory, X-ray, *etc.*) data loss or retransmissions could occur if protocol is not designed with mobility support.
  d) External factors such as a monitoring environment with heavy traffic information (external devices are transmitting information) or geographic areas that prevent proper communication between network devices.
  e) The need to guarantee access to the transmission channel for each sensor according to the priority that involves each of the scenarios for monitoring.
4. *Quality of service*: It is of utmost importance in the monitoring and vital sign transmission; data must be available at the right time, without errors and in real time, so reliability of the network is not affected.
5. *Security:* The security and privacy of patient-related data are two relevant components for the system security of the WBSN. By data security, it means the protection of information from unauthorized users while data being stored and transferred and data privacy means right of individuals to control the collection and use of personal information. Security and privacy issues are raised automatically when the data is created, transferred, stored and processed. The Health Insurance Portability and Accountability Act (HIPAA) mandates that, as the sensors in WBSN collect the wearer’s health data (which is regarded as personal information), care needs to be taken to protect it from unauthorized access and tampering [27].
6. *Critical time parameters:* For diagnosis, it is very important that health data (vital signs) of patients arrive within the expected time to accurately indicate the actual health condition of the patient. Delay, packet errors, and packet losses are some factors that could alter the correct medical diagnosis. If one of the vital sign parameters gets lost, arrives with delay or in a different time slot, a critical condition could be ignored. So, these factors are needed to evaluate a WBSNs through critical time parameters [60].

This research has presented information to develop an adaptive system optimization. This is important because the energy consumption could be altered by multiple factors when the monitoring is conducted in a patient, e.g., it is not the same to monitor three patients as eight, two or five vital signs or to carry out the WBSN monitoring in a rural area as in an urban area with 4G cell phone network. It is necessary to take into account that each patient’s condition represents different needs and priorities to evaluate, in other words, there is a big difference of patients being monitored in an emergency situation from that of inside hospitals or in their homes. By developing a system which is sensitive to changes of variables such as polling interval, traffic generated by sensors, priorities for monitoring or energy remaining in the system, we will make a significant contribution towards improving performance to maximize the WBSN lifetime. The aim of the development for this proposal is based on contributing in the area of telemedicine that seeks to provide better care to patients in each of the above scenarios allowing a more reliable doctor-patient relationship. Having a WBSN with high performance and in real-time, we can improve the disease control practices resulting in the improvement of patient health status.

In terms of network optimization for the transmission of data and video over limited bandwidths, that is common for m-health scenarios, it is necessary to reduce the required resources from the network. A common approach to minimize the physical data channels of the network is the joint-transmission of different elementary streams like: video, audio, physiological signals, personal data, *etc.* The joint-transmission of the streams can be achieved via multiplexing, that is common for an audio/video streaming in a media context. 

Recently, data hiding techniques have been implemented for media context, like those based on different block sizes taking the rate-distortion into account to achieve good performance in the audio-video synchronization, one stream of information (audio) embedded into the video signal [61,62]. The advantages envisioned for this technique over multiplexing, resides in the protection/privacy of the medical data and the natural synchronization achieved between the elementary streams and the video. With data hiding, the transmission of medical data (physiological signals, personal data) will be embedded into the video stream, so only one communication channel is needed, moreover the privacy and accurate synchronization is established. Whenever the frequency of the physiological signals is greater than the frequency encoded video sequence capacity, data compression can be necessary to overcome the visualized data hiding synchronization scheme. Therefore, high frequency compressed signals could be hidden into the video signal with accurate synchronization and with less quality degradation of video caused by the data hiding scheme. 

## 8. Conclusions

In this survey we have reviewed the state of the art of WBSN providing a wide overview of features, practices and the most important issues that researchers will face for emergency scenarios. Unlike other surveys we have presented information that helps to contemplate development of an adaptive system optimization according to the needs and behavior of the network at any given moment. The development of the proposed system would allow extending its application to diverse scenarios as patients with chronic-degenerative illnesses, preventive monitoring or patients in intensive care. Additionally, we consider the video transmission as an important feature into a WSBN in an emergency scenario. The visual interaction between the paramedic and the doctor in this context is very helpful to enhance the medical treatment of the victim. We introduce some aspects like the video and vital sign joint-transmission in real time, and the synchronization between them, supporting the physicians to have a better picture of patient’s condition in order to have a better diagnosis. However, our research helps us to understand the existence of some issues ignored, for example, in the MEDISN [17], the coexistence between different technologies and noise signals that can be found inside a building is not evaluated, especially if network is deployed with multiple sensors of different transfer rates struggling to send information. Similarly, it shall be verified the performance under network scalability conditions.

Nevertheless, situations as mobility of sensors, wireless technology coexistence and noise are not addressed. So in addition with the above issues the following question arises: is it possible that by using different technologies applied in WBSN, communication protocols and capabilities of network devices, a reduction of energy consumption can be achieved? Surely it can be attained, but it will be necessary to develop new schemes and methodologies that allow analyzing and optimizing the exchange of information between the devices. Strategies like these will help to determine the characteristics that a model or system must have within the framework of energy efficiency. Once the last question is resolved, patients could increase their survival possibilities because they will have specialized medical care starting at the scene of the accident or emergency event.
